# Middle Meningeal Artery Embolization in the Treatment of Chronic Subdural Hematoma: A Two-Center Retrospective Study

**DOI:** 10.3390/jcm14228226

**Published:** 2025-11-20

**Authors:** Francesco Adduci, Bruno Del Sette, Giancarlo Salsano, Greta Venturi, Carmelo Sturiale, Massimo Dall’Olio, Claudia Rolla Bigliani, Pietro Fiaschi, Luigi Cirillo, Lucio Castellan

**Affiliations:** 1 Department of Experimental, Diagnostic and Specialty Medicine (DIMES), University of Bologna, 40138 Bologna, Italy; francesco.adduci@studio.unibo.it; 2 Department of Neuroradiology, IRCCS Ospedale Policlinico San Martino, 16132 Genova, Italy; giancarlo.salsano@hsanmartino.it (G.S.); claudia.rollabigliani@hsanmartino.it (C.R.B.); lucio.castellan@hsanmartino.it (L.C.); 3 Functional and Molecular Neuroimaging Unit, IRCCS Istituto delle Scienze Neurologiche di Bologna, 40139 Bologna, Italy; greta.venturi@studio.unibo.it (G.V.); carmelo.sturiale@ausl.bologna.it (C.S.); massimo.dallolio@aosp.bo.it (M.D.); luigi.cirillo2@unibo.it (L.C.); 4 UOC di Neuroradiologia, IRCCS Istituto delle Scienze Neurologiche di Bologna, 40124 Bologna, Italy; 5 Division of Neurosurgery, IRCCS Ospedale Policlinico San Martino, 16132 Genova, Italy; pietro.fiaschi@hsanmartino.it; 6 Department of Neurosciences, Rehabilitation, Ophtalmology, Genetics, Maternal and Child Health (DI-NOGMI), University of Genova, 16132 Genova, Italy; 7 Department of Biomedical and Neuromotor Sciences (DIBINEM), University of Bologna, 40126 Bologna, Italy

**Keywords:** cSDH, MMA, embolization, Markwalder score, hematoma thickness reduction, EVOH, trabecular subtype

## Abstract

**Background**: Chronic subdural hematoma (cSDH) is a common condition, particularly in individuals over 65 years of age. Its pathophysiology involves traumatic and inflammatory processes, culminating in hematoma formation. Although surgical drainage is the primary treatment of choice, its significant recurrence rates have prompted exploration of non-surgical options. This study evaluates the effectiveness of middle meningeal artery (MMA) embolization as an alternative or adjunctive treatment in asymptomatic or paucisymptomatic cSDH patients. **Methods**: This two-center retrospective study analyzed 93 patients treated with MMA embolization at two hospitals between 2020 and 2024. Patients exhibited either asymptomatic or mild symptomatic cSDH (Markwalder score 0 or 1), with hematoma thickness >10 mm. Pre- and post-treatment CT scans were compared to assess hematoma thickness, with follow-ups conducted at three months. Statistical analysis included ANCOVA and Mann–Whitney U tests for outcome evaluation. **Results**: Of the 93 patients, 44 underwent bilateral embolization, and 49 underwent unilateral embolization. Treatment reduced hematoma thickness by an average of 59% at three months follow-up. The trabecular subtype showed the greatest thickness reduction compared to other subtypes (*p* < 0.05). No significant differences were found between embolization materials. Only 3.2% of patients experienced technical complications, and three patients experienced rebleeding during follow-up. **Conclusions**: MMA embolization appears to be a safe and effective treatment for cSDH, providing a minimally invasive alternative to surgery. The technique shows promise in reducing hematoma size, especially in the trabecular subtype. Further research is needed to confirm these findings and establish their role in routine clinical practice.

## 1. Introduction

Chronic subdural hematoma (cSDH) is a common neurological condition, with an estimated incidence of approximately five cases per 100,000 people, which is significantly higher in individuals over 65 years of age [1]. The pathophysiology of cSDH is complex and not fully understood. Initially, it was thought to result mainly from traumatic injury, leading to the rupture of the bridging veins. However, recent evidence highlights the pivotal role of chronic inflammatory processes within the subdural space. Some studies suggest that injury to the capillary-rich dural border cell layer, located at the dura–arachnoid interface, initiates a chronic inflammatory response that sustains vascular leakage and gradual hematoma formation. In addition, blood accumulation triggers an inflammatory cascade, characterized by the release of cytokines, chemokines, reactive oxygen species, and angiogenic factors, promoting the development of neovascularized inflammatory membranes in the subdural compartment [2,3,4]. Surgical evacuation, typically performed through burr-hole drainage or craniotomy, remains the standard first-line therapy for symptomatic patients, as it rapidly alleviates the mass effect. Despite being the mainstay of cSDH management, burr-hole drainage and craniotomy carry notable recurrence and complication rates. Recurrence occurs in approximately 10–37% of cases, depending on patient age, comorbidities, and anticoagulant/antiplatelet therapies, often necessitating repeat intervention. Reported postoperative complication rates range between 5 and 20%, including infection, seizures, acute rebleeding, and, less frequently, postoperative tension pneumocephalus. These risks are particularly pronounced in elderly patients and those requiring early resumption of anticoagulant or antiplatelet therapy, underscoring the need for safer, minimally invasive alternatives such as MMA embolization. Furthermore, the need for this patient population to very often quickly restart anticoagulant and antiplatelet therapy has pushed the trend towards the identification of a non-surgical endovascular treatment. Conservative management with corticosteroids or statins has been explored, but it remains controversial and lacks robust evidence of efficacy [5]. In response to these challenges, growing attention has been directed toward alternative strategies, particularly MMA embolization. In recent years, the rationale for embolization has emerged from a better understanding of cSDH pathophysiology—specifically, the role of fragile neovascularized membranes fed by MMA branches. Embolization aims to interrupt this vascular supply, promoting membrane regression and hematoma resorption while minimizing surgical risks. This minimally invasive endovascular procedure targets the inflammatory cascade within pathological membranes, reducing capillary bleeding and promoting hematoma resolution. This therapeutic rationale supports the exploration of MMA embolization as a primary or adjunctive strategy in selected patients. The aim of our study is to evaluate the effectiveness of endovascular MMA embolization in asymptomatic or paucisymptomatic patients, as an alternative or adjunct to surgical treatment.

## 2. Materials and Methods

### 2.1. Study Design and Patient Selection

**Figure 1:**
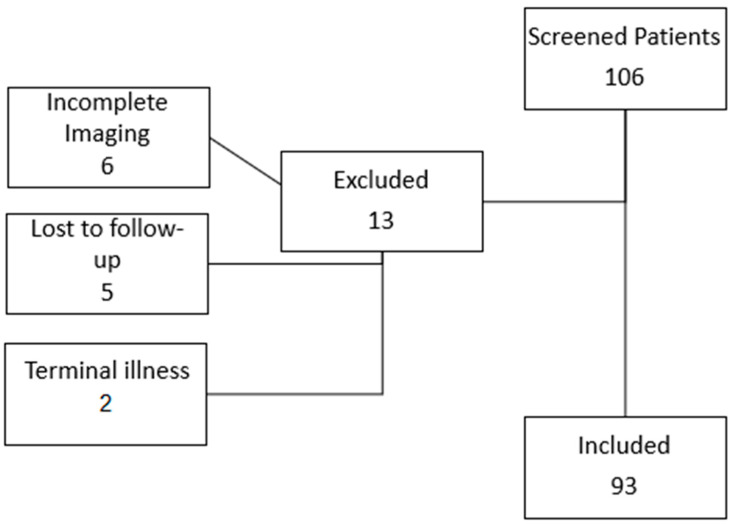
Flow diagram of included patients.

This two-center retrospective cohort study includes all consecutive patients with cSDH treated by MMA embolization between January 2020 and May 2024 at the IRCCS San Martino Polyclinic Hospital in Genova (66 patients) and the IRCCS Institute of Neurological Sciences in Bologna–Bellaria Hospital (27 patients). Inclusion criteria were age ≥ 18 years, asymptomatic or mildly symptomatic cSDH (Markwalder grading scale 0–1), thickness of >10 mm, and availability of baseline and follow-up CT scans. Exclusion criteria included life expectancy <6 months; ventricular shunts, contraindications to angiographic examination (e.g., vascular anatomy preventing safe access, or terminal renal failure). Of 106 screened patients, 13 were excluded (six incomplete imaging, five lost to follow-up and two because of terminal illness), leaving 93 for analysis (Figure 1).

The therapeutic indication for MMA embolization was established through the multidisciplinary consensus between neurosurgeons and interventional neuroradiologists at each institution. Patients were considered eligible if imaging demonstrated a hematoma thickness of >10 mm and a midline shift of <8 mm, even in the absence of significant neurological symptoms, provided there was a risk of progression due to anticoagulant or antiplatelet therapy, presence of hyperdense components within the collection, suggesting the presence of an immature membrane, prone to bleeding, or failure of spontaneous resolution during radiological follow-up.

Indications for MMA embolization in patients not receiving antiplatelet or anticoagulant therapy were primarily radiological and preventive. Patients with large collections (>10 mm) with hyperdense components or those demonstrating poor spontaneous resolution in the follow-up imaging were considered for prophylactic embolization to prevent potential expansion or symptom progression. In these cases, the procedure was viewed as an opportunity to avoid future surgery in otherwise frail or elderly patients.

Regarding the laterality of the embolization, the choice between unilateral and bilateral procedures was based on hematoma distribution and angiographic findings. Bilateral embolization was performed in cases of bilateral cSDH or when both middle meningeal arteries contributed to the vascularization of the hematoma membranes, as demonstrated by selective angiography. In strictly unilateral collections with no significant contralateral contribution, embolization was limited to the affected side. This individualized approach reflects the variability of MMA anatomy and collateral supply patterns.

All patients provided written informed consent prior to the procedure, and the study was conducted in accordance with the principles of the Declaration of Helsinki. The study protocol received approval from the institutional ethics committees of IRCCS Ospedale Policlinico San Martino (Genova, Italy) and IRCCS Istituto delle Scienze Neurologiche di Bologna–Bellaria Hospital.

### 2.2. Embolization Procedure

Procedures were standardized across centers using transfemoral access, using a 5 or 6 French vascular sheath and performing digital subtraction angiography of the external and internal carotid arteries to assess the anatomy of the MMA, dangerous collaterals, and anatomical variations. Selective catheterization of the MMA was performed using microcatheters (0.014–0.017 inch) for distal embolization into the fronto-parietal and squamo-temporal branches. The embolic materials included EVOH (Onyx/Squid), PVA, PVA + EVOH, PVA + coils, or Glubran. The choice of embolic material evolved over time, according to institutional availability, operator preference, and anatomical features of MMA, reflecting real-world practice. PVA were preferred in tortuous and difficult-to-navigate arteries, whereas EVOH were usually selected for straight arteries, which were easier to navigate distally to achieve the wedge position of the microcatheter.

### 2.3. Data Collection and Analysis

All patients were clinically and laboratory analyzed, assessing their comorbidities and coagulation status (whether on antiplatelet or anticoagulant therapy), although these characteristics were not a condition for inclusion or exclusion from treatment. Pre- and post-treatment CT characteristics were examined, with a 3 to 6-month follow-up, focusing on subdural layers’ thickness reduction as the main outcome. Radiological evaluations were performed independently by two blinded neuroradiologists (G.S and B.D.S) to minimize bias. For each patient, the following data were identified: laterality of onset of the subdural hematoma (unilateral or bilateral) and if unilateral, the site of onset (right or left); hematoma architecture subtypes, as described by Nakaguchi et al. [6] (homogeneous, laminar, separate, and trabecular), thickness of the pre- and post-treatment cSDH, presence of midline shift pre- and post-embolization, density of the cSDH pre- and post-treatment and rebleeding episodes during follow-up Angiographic images were analyzed and we identified indication for embolization (post-surgical or first treatment); laterality of the treatment (unilateral or bilateral); embolizing material used (Ethylene-vinyl alcohol copolymer-based liquid embolic agent (EVOH), polyvinyl acetate beads (PVA), PVA + EVOH, PVA + coils, Glubran); and technical procedural complications and failures.

The primary endpoint was the percentage reduction in the maximal hematoma thickness at 3 months, measured on CT on the coronal plane at the slice showing maximal width. A successful radiological response was predefined as reduction ≥ 50%. Secondary endpoints included rebleeding, surgical rescue, changes in the Markwalder scale, mortality, and technical/clinical complications.

### 2.4. Statistical Analysis

Data distribution normality was assessed using the Shapiro–Wilk Test. Statistical analyses were conducted using ANCOVA to compare normal values and linear regression prior to the Mann–Whitney U test for non-normal values. Because of the sample size, multivariable regression was not performed, but exploratory subgroup analyses (age, laterality, subtype, and anticoagulation) were examined. *p* < 0.05 was considered significant.

## 3. Results

A total of 93 patients, with a mean age of 75.49 years, were included in the study. Patients’ characteristics are summarized in Table 1. Among them, 44 patients underwent bilateral intervention, while 49 underwent unilateral intervention, treating a total of 137 treated hematomas. At the time of presentation, 37.6% were on antiplatelet therapy, 23.5% were on anticoagulant therapy, and 38.9% were not on any of those medications. Endovascular treatment was performed as first-line therapy in 64.5% of cases, while in 35.5%, it was conducted post-surgically. Only three patients (3.2%) could not complete the treatment during the procedure, due to excessive tortuosity of the epiaortic vessels, which led to inability to catheterize the external carotid artery and the MMA; another three patients (3.2%) completed only a unilateral treatment instead of the planned bilateral intervention (due to stenosis at the origin of the MMA in one case and origin of the ophthalmic artery from the MMA in the other two). Only one patient (1.1%) experienced clinical complications, with transient left-sided hemiparesis without vascular occlusion and symptom regression after 24 h; patient mortality was 0%. Of the 90 patients who completed the treatment, 130 MMA embolizations were performed using various materials: EVOH 33.9%, PVA 20.8%, PVA + EVOH 23.8%, PVA + coils 20.8%, and Glubran 0.7%. For PVA, 150–250 µm particles were used in 62 cases out of 85, while for the other 23, the 250–355 µm size was preferred; for EVOH agents, 0.3–0.7 mL per branch was injected under continuous fluoroscopic control until occlusion. There was no significant difference in outcomes between the different embolization materials (*p* > 0.05) (Table 2). Follow-up imaging using CT (mean 6.2 ± 2.6 months) showed an average reduction in the cSDH thickness of approximately 59% at three months (SD 33%). Radiological success, defined as hematoma reduction >50% was achieved in 64.2% of the cSDH (88/137), while complete hematoma resolution was registered for 34.3% (47/137). Midline shift was reduced in 22 out of the 27 patients that presented it at the pre-intervention CT scan; two remained stable, while only three patients presented a significant increase in the thickness of the subdural collection at follow-up, and two of them required surgical evacuation due to the development of neurological symptoms and the development of midline shift >1 cm.

No clinical changes were witnessed in the other 91 patients with stability of the Markwalder grade. Among these 93 patients, 137 hematoma collections (64 right, 73 left) were evaluated and categorized by subtypes described by Nakaguchi: 51 homogeneous type, 31 laminar type, 14 separated type, and 41 trabecular type. Post-embolization analysis revealed that the trabecular subtype showed a statistically significant greater reduction in thickness compared to laminar and separated subtypes (*p* = 0.03). No statistically significant difference in outcomes was witnessed between post-surgical patients and first-line patients (*p* > 0.05).

## 4. Discussion

### 4.1. Treatment Effectiveness

**Figure 2:**
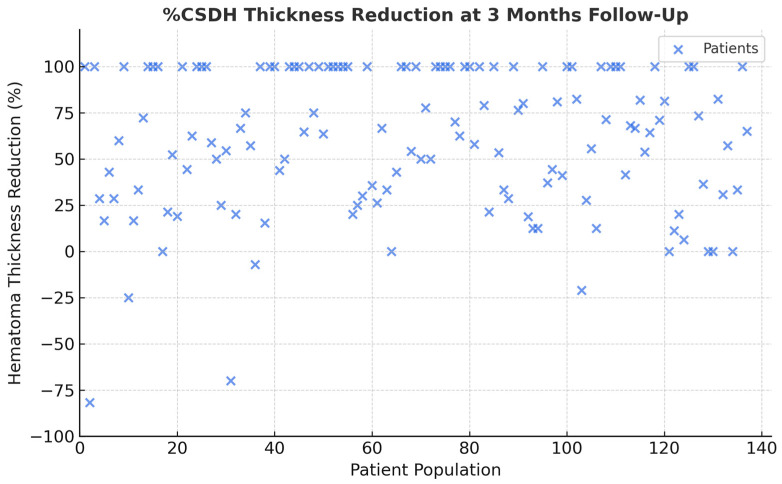
Scatter plot: the graph illustrates the distribution of the percentages of reduction in the thickness of the cSDH after the treatment. It indicates that in more than a third of cases, there was a 100% resolution at three months.

Our exploratory two-center retrospective analysis demonstrated that MMA embolization achieved a significant reduction in hematoma thickness at 3 to 6 months, with a low rate of complication and no long-term morbidity related to the procedure (Figure 2). These findings are consistent with the growing body of literature supporting MMA embolization as an effective adjunct or alternative treatment modality in asymptomatic patients with cSDH [7].

The main limitation of this study lies in the absence of a control group treated conservatively or surgically, as well as the retrospective design of it. This makes it impossible to exclude that a proportion of the observed hematoma reduction might have occurred spontaneously, particularly in asymptomatic patients. Several previous series have demonstrated that up to one-third of small, stable, or trabecular-type cSDHs may undergo partial or complete resorption without active intervention [8]. Therefore, while our findings show a marked mean reduction following embolization, these results should be interpreted with caution and seen as exploratory. The evidence supports the biological plausibility of MMA embolization promoting resolution, but spontaneous evolution cannot be ruled out without controlled comparative data.

**Figure 3:**
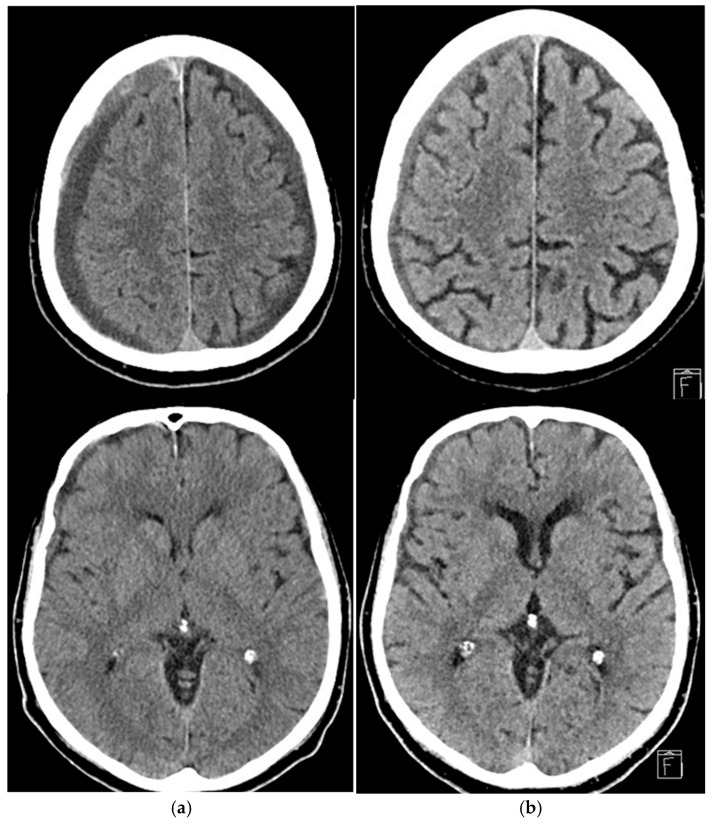
Representative case: 76-year-old male on antiplatelet therapy, presenting with bilateral chronic subdural hematoma (**a**) homogeneous hypodense subtype on the left, with side maximum thickness of 10 mm and trabecular subtype on the right side, with maximum thickness of 14 mm. Embolization with EVOH achieved >50% reduction in thickness at 3 month follow-up, which was more evident at the vertex, with concurrent re-expansion of the cerebral sulci, with full clinical recovery (**b**). No adverse events were reported.

Notably, Kan et al. treated patients with first-time cSDH and with recurrence after prior evacuation, and reported similar results, having obtained a primary outcome of 50% decrease in hematoma thickness in 70.8% of lesions, within an average follow-up of 94.9 days [9]. Similarly, Link et al. expanded on this evidence by treating patients across a spectrum of different clinical scenarios, including patients with previously untreated cSDH, with recurrent cSDH after surgical evacuation, or as prophylactic treatment, immediately after surgical evacuation. They reported a reduction of >50% in the maximum thickness of cSDH in 72.0% of cases at a follow-up of at least 6 weeks (Figure 3) [10]. These results suggest that endovascular embolization of MMA can induce hematoma resolution effectively and in a relatively short period.

Despite these promising outcomes, the role of MMA embolization in treating chronic subdural hematoma is still much debated. Several studies have shown the value of treatment in reducing the rate of recurrence of bleeding [11,12]. For instance, Ban et al. demonstrated that out of 72 patients undergoing embolization surgery, only one patient experienced a bleeding recurrence, following additional head trauma [13], underlining how endovascular treatment carries a protective effect against spontaneous rebleedings. The role of embolization of the subdural hematoma has been studied both in association with and as an alternative to surgery, obtaining very promising results [14,15]. Traditional treatment of symptomatic patients with cSDH has always been surgical evacuation, which, while effective in reducing the hematoma and therefore the neurological symptoms, still harbors a significant risk of recurrence—up to 30%—which may require subsequent interventions. Several studies have reported that systematic postoperative endovascular treatment can significantly reduce recurrence rates, in some cases achieving rates as low as 0% during follow-up periods of up to six months. [16,17,18]. This suggests that endovascular embolization may target the pathophysiological process of cSDH, such as the development of immature neomembranes, allowing for a more stable post-surgical result. Currently, the role of endovascular treatment as first line, without surgery, is debated, and some studies have already shown excellent results. A retrospective study by Onyinzo suggests that embolization, both as a standalone treatment or in addition to surgery, offers better outcomes and facilitates quicker resumption of anticoagulant or antiplatelet therapy: a crucial point in managing patients with underlying cardiovascular comorbidities [19]. Comparatively, the role of MMA embolization versus conservative medical treatment is yet to be fully established. In this regard, the first results appear comforting; a retrospective analysis by Marulanda et al. showed a trend for lower recurrence and mortality rates in the embolization era cohort, with better radiological outcome at follow-up, but with no significant differences in complications and clinical improvement [20]. On the other hand, the recent results of the EMPROTECT trial showed no significant difference in recurrence rates at six months between MMA embolization and standard medical therapy in patients previously undergoing surgery with a high risk of recurrence, therefore not showing a clear superiority of embolization [21]. In a similar way, the MAGIC-MT trial, one of the largest RCTs conducted to date, failed to demonstrate a statistically significant superiority of embolization compared with standard conservative treatment, in terms of reducing recurrence or hematoma progression. However, the study did identify a lower rate of serious adverse events in the embolization group compared with standard therapy (6.7% vs. 11.6%; *p* = 0.02) [22]. This suggests that while embolization may not consistently translate into a reduction in recurrence rates when compared directly with conservative treatment, it may nonetheless offer a safety benefit that could be clinically meaningful, particularly in vulnerable patient populations. All these findings highlight the complexity of cSDH pathophysiological processes and suggest that MMA embolization remains investigational, particularly for asymptomatic patients. Careful patient selection should be critical in this population, considering comorbidities and risk of worsening. Our study showed a greater reduction in the thickness of the subdural hematoma in its trabecular subtype, suggesting that it may respond more favorably to embolization due to the greater presence of membranes within it, which are ideally the target of MMA embolization. Due to this assumption, we preferred to inject the embolizing agents as distally as possible to allow a better penetration into the neomembranes, maximizing this effect. However, this finding contradicts the results of the multicenter study by Weinberg et al., which found no correlation between membranes’ presence and recurrence or retreatment rates, suggesting that the relationship between hematoma architecture and treatment response remains a complex and not fully understood process [23]. One plausible explanation is that the trabecular subtype represents a later, more organized stage of the hematoma, with reduced neovascular fragility, in which embolization effectively induces gradual resorption rather than preventing recurrent bleeding. This observation suggests that MMA embolization may be particularly effective as a stabilizing or adjuvant therapy in selected trabecular cases, especially following surgery or in patients with limited physiological reserve. Further histopathological correlation and prospective validation would be valuable to clarify this relationship.

### 4.2. Embolic Materials: Advantages and Limitations

An unresolved and still crucial aspect in the field of MMA embolization for chronic subdural hematoma concerns the choice of embolic material. In our experience, different agents have been employed, yet none has demonstrated clear superiority in terms of clinical efficacy. In the literature, the most frequently used liquid embolic agents are Onyx™ (Medtronic, Dublin, Ireland) and Squid™ (Balt, Montmorency, France). Both are non-adhesive liquid polymers, composed of ethylene vinyl alcohol (EVOH) dissolved in dimethyl sulfoxide (DMSO), rendered radiopaque by the addition of metallic particles. These agents have largely replaced solid particles, such as polyvinyl alcohol (PVA), because of their ability to penetrate more distally into the vascular branches, theoretically allowing for a more complete and durable embolization. Several randomized controlled trials (RCTs) have investigated the performance of these liquid agents. The OTEMACS trial emphasized that Onyx™ provides the advantage of permanent radiopacity, enabling real-time visualization of its distribution during the procedure and reducing the risk of reflux into non-target vessels [24]. Similarly, the STEM trial demonstrated the feasibility and safety of Squid™, without any significant increase in adverse events [14]. In addition, another embolization technique has been described, in which proximal coiling is combined with the injection of liquid agents, with the intent of reinforcing the embolization and achieving a more stable occlusion [25]. Despite these technical differences, none of the available RCTs has shown the clinical superiority of one embolic material over another [26]. At present, Onyx™, Squid™, PVA microparticles, and proximal coiling techniques should all be regarded as valid and safe options. The choice among them is often guided by operator preference, local availability, and individual case characteristics, rather than by evidence of differential clinical efficacy (Table 3) [27].

### 4.3. Study Limitations and Future Directions

While our results are promising, several limitations must be acknowledged. As previously stated, the retrospective, non-randomized design and the absence of a control group prevent direct comparison with surgical or conservative treatments. Potential selection and information biases may have influenced the findings, as only patients undergoing embolization were included. Minor procedural variations and incomplete data also represent possible confounders, despite efforts to standardize protocols and employ blinded radiological assessments to reduce variability.

Nevertheless, the study identifies potential radiological predictors—such as the trabecular subtype—that may respond more favorably to embolization, underscoring the importance of individualized patient selection. Future multicenter, prospective randomized trials are warranted to validate these results, optimize procedural techniques, and better define the role of middle meningeal artery embolization in the comprehensive management of chronic subdural hematoma.

Another practical aspect concerns the cost and resource implications of MMA embolization. The procedure involves specialized materials (microcatheters, liquid embolic agents), dedicated angiographic suite time, and at least short-term post-procedural monitoring, resulting in higher upfront costs than conservative management [28]. However, this must be balanced against the potential savings from reduced recurrence rates, avoidance of surgical re-interventions, and shorter hospital stays. A formal cost-effectiveness analysis was beyond the scope of this retrospective study, but future prospective comparisons, including conservative management for asymptomatic, non-anticoagulated patients, are warranted to establish the economic sustainability of embolization as a first-line therapy.

## 5. Conclusions

In conclusion, our findings from this double-center retrospective analysis add to the growing evidence supporting MMA embolization as valuable tool in the management of cSDH patients. MMA embolization achieved a 59% mean hematoma thickness reduction, with a low complication rate and no significant difference between embolic materials. The technique appears to be particularly effective for trabecular subtypes, suggesting possible morphological predictors of success. These results reinforce embolization as a viable, minimally invasive alternative in selected cSDH patients, warranting validation through prospective trials. While this therapeutic alternative shows promise in providing hematoma reduction and lower recurrence rates, the heterogeneity of patient population, hematoma subtypes, and study methodologies requires further clinical trials. Future multicenter randomized trials comparing MMA embolization with burr-hole drainage or conservative management are necessary. Further investigations should clarify optimal timing, embolic material, and patient profiles that are most likely to benefit from endovascular therapy.

## Figures and Tables

**Table 1:** Patient demographics and clinical characteristics (*n* = 93).

Variable	Value
Mean age (years)	75.5 ± 8.9
Sex (male/female)	61/32
Hypertension	58 (62.3%)
Diabetes	17 (18.3%)
Cardiovascular disease	22 (23.6%)
Antiplatelet therapy	35 (37.6%)
Anticoagulant therapy	22 (23.5%)
None	36 (38.9%)
First-line embolization	60 (64.5%)
Post-surgical embolization	33 (35.5%)
Bilateral/unilateral	44/49
Midline shift present	27 (29%)
Markwalder 1	63 (67.7%)

**Table 2:** Type of embolization materials for each individual side of cSDH, with respective radiological results and complications.

Embolization Materials	cSDH (%)	Mean Thickness Reduction (%) ± SD (*p* > 0.05)	Clinical Complications	Rebleeding
EVOH	44 (33.9)	59.2 ± 33.1	0	1
PVA	27 (20.8)	58.6 ± 31.7	1 (transient deficit)	1
PVA + EVOH	31 (23.8)	61.3 ± 32.9	0	0
PVA + COIL	27 (20–8)	57.9 ± 34.2	0	1
GLUBRAN	1 (0.7)	0	0	0

**Table 3:** Comparative summary table of different embolic agents.

Material	Type	Main Advantage	Main Limitation
EVOH	Liquid	Deep distal penetration	Cost, DMSO irritation
PVA	Particulate	Widely available	Less durable occlusion
Coils	Mechanical	Stable occlusion	Proximal only, incomplete distal flow block

## Data Availability

Data will be made available upon request.

## Abbreviations

cSDH	Chronic Subdural Hematoma
MMA	Middle Meningeal Artery
PVA	Polyvinyl Acetate
EVOH	Ethylene Vinyl Alcohol Copolymer-Based Liquid Embolic Agent
